# 1,3,5-Tri-*p*-tolyl­pentane-1,5-dione

**DOI:** 10.1107/S1600536808036854

**Published:** 2008-11-13

**Authors:** You-Liang Shen

**Affiliations:** a Jiangxi Key Laboratory of Surface Engineering, Jiangxi Science and Technology Normal University, Jiangxi 330013, People’s Republic of China

## Abstract

In the crystal structure of the title compound, C_26_H_26_O_2_, the dihedral angle between the tolyl rings at each end of the 1,5-dione chain is 70.3 (1)°; the tolyl group at the middle of the chain makes dihedral angles of 67.8 (2) and 85.1 (2)° with the terminal rings. One benzene C atom and one methyl­ene C atom inter­act with a carbonyl O atom of an adjacent mol­ecule through C—H⋯O hydrogen bonds, forming chains in the crystal.

## Related literature

For the details of related structures, see: Burroughes *et al.* (1990 ▶); Smith *et al.* (2005 ▶); Li *et al.* (2004 ▶); Sariciftci *et al.* (1992 ▶). For the synthesis of the title compound, see: Yang *et al.* (2005 ▶).
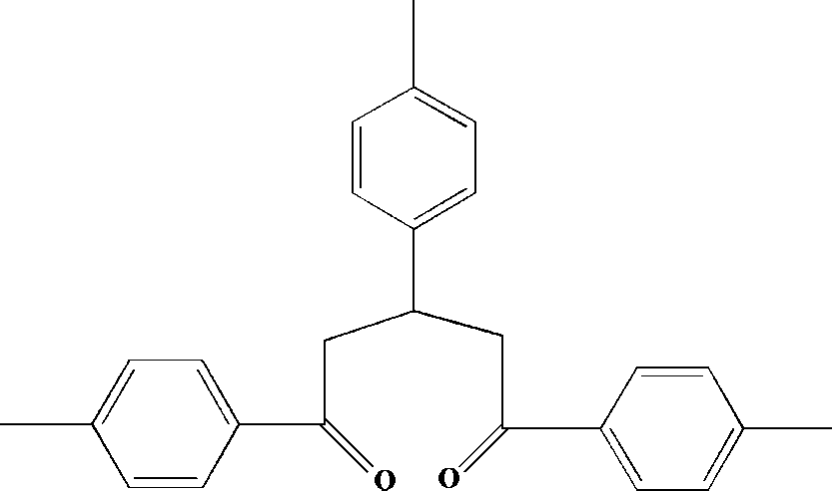


## Experimental

### 

#### Crystal data

C_26_H_26_O_2_
*M* *_r_* = 370.47
Orthorhombic, 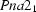

*a* = 10.6611 (19) Å
*b* = 10.3876 (18) Å
*c* = 19.541 (3) Å
*V* = 2164.0 (6) Å^3^
*Z* = 4
Mo *K*α radiation
μ = 0.07 mm^−1^
*T* = 295 (2) K
0.34 × 0.24 × 0.18 mm

#### Data collection

Bruker SMART APEX area-detector diffractometer
Absorption correction: multi-scan (*SADABS*; Sheldrick, 1996 ▶) *T*_min_ = 0.977, *T*_max_ = 0.991
8705 measured reflections
2138 independent reflections
1733 reflections with *I* > 2σ(*I*)
*R*_int_ = 0.031

#### Refinement

*R*[*F* ^2^ > 2σ(*F* ^2^)] = 0.057
*wR*(*F* ^2^) = 0.135
*S* = 1.06
2138 reflections
256 parameters
1 restraint
H-atom parameters constrained
Δρ_max_ = 0.14 e Å^−3^
Δρ_min_ = −0.12 e Å^−3^

### 

Data collection: *SMART* (Bruker, 2002 ▶); cell refinement: *SAINT* (Bruker, 2002 ▶); data reduction: *SAINT*; program(s) used to solve structure: *SHELXS97* (Sheldrick, 2008 ▶); program(s) used to refine structure: *SHELXL97* (Sheldrick, 2008 ▶); molecular graphics: *SHELXTL* (Sheldrick, 2008 ▶); software used to prepare material for publication: *SHELXTL*.

## Supplementary Material

Crystal structure: contains datablocks I, global. DOI: 10.1107/S1600536808036854/wn2290sup1.cif

Structure factors: contains datablocks I. DOI: 10.1107/S1600536808036854/wn2290Isup2.hkl

Additional supplementary materials:  crystallographic information; 3D view; checkCIF report

## Figures and Tables

**Table 1:** Hydrogen-bond geometry (Å, °)

*D*—H⋯*A*	*D*—H	H⋯*A*	*D*⋯*A*	*D*—H⋯*A*
C7—H7⋯O2^i^	0.93	2.46	3.381 (5)	171 (1)
C18—H18*A*⋯O2^i^	0.97	2.52	3.460 (5)	164 (1)

Symmetry code: (i) 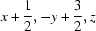.

**Fig. 1.**
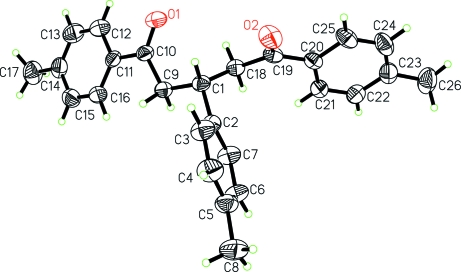
The molecular structure, with the displacement ellipsoids drawn at the 30% probability level. The H atoms are shown as spheres of arbitary radii.

**Fig. 2.**
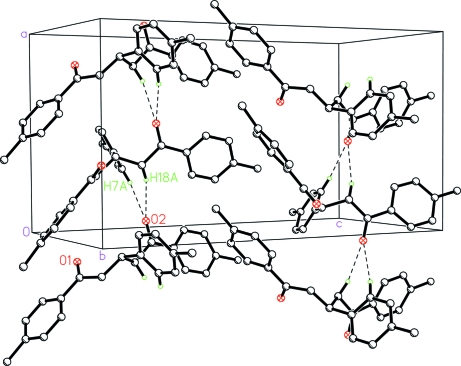
A packing diagram of the title structure, showing the intermolecular C—H···O hydrogen bonds as dashed lines. The H atoms not involved in hydrogen bonding have been omitted for clarity.

## supplementary crystallographic information

### Comment

Over the past several decades, linear π-conjugated organic molecules and polymers have attracted considerable interest because of their many promising applications, such as for organic light-emitting diodes, non-linear optical properties, conductivity, photocells, field-effect transistors, and so on, due to their delocalized π systems (Burroughes *et al.*, 1990; Smith *et al.*, 2005; Li *et al.*, 2004; Sariciftci *et al.*, 1992). In the course of our synthesis of the π-conjugated organic molecule, 2,4,6-tri-*p*-tolyl-pyridine, we synthesized the 1,5-dione intermediate 1,3,5-tri-*p*-tolyl-pentane-1,5-dione; the 1,5-dione intermediate was then cyclized by adding concentrated aqueous ammonia. We report here the crystal structure of the 1,5-dione intermediate, 1,3,5-tri-*p*-tolyl-pentane-1,5-dione.

As shown in Fig. 1, the title molecule is non-planar, and the dihedral angles between each pair of the three tolyl rings are 67.8 (2)° [C2–C7, C11–C16], 70.3 (1 ° [C11–C16, C20–C25] and 85.1 (2)° [C2–C7, C20–C25]. The C—C, C_ar_—C_ar_ and C═O bond lengths are within their normal ranges. One benzene C atom (C7) and one methylene C atom (C18) interact with a carbonyl group O atom (O2) of an adjacent molecule through C—H···O hydrogen bonds [3.381 (5) Å, 3.460 (5) Å] to form a one-dimensional supramolecular array (Fig. 2).

### Experimental

The title compound was synthesized according to a modified procedure (Yang *et al.*, 2005). 4-Methylacetophenone (0.5 g, 4 mmol), 1,3-di-*p*-tolyl-propenone (0.9 g, 4 mmol) and powdered NaOH (0.6 g, 15 mmol) were crushed together for 2 h, using a pestle and mortar. Recrystallization from ethanol gave colorless prismatic crystals. Yield: 1.2 g (88%).

### Refinement

All H-atoms were positioned geometrically and refined using a riding model with C—H = 0.93 Å, 0.97 Å , 0.98 Å, *U*_iso_(H) = 1.2*U*_eq_(C) for aromatic, methylene and methine H atoms; 0.96 Å, *U*_iso_(H) = 1.5*U*_eq_(C) for methyl groups. In the absence of significant anomalous scattering effects, the Friedel pairs were merged.

### Crystal data

**Table:**

C_26_H_26_O_2_	*F*_000_ = 792
*M**_r_* = 370.47	*D*_x_ = 1.137 Mg m^−^^3^
Orthorhombic, *P**n**a*2_1_	Mo *K*α radiation λ = 0.71073 Å
Hall symbol: P 2c -2n	Cell parameters from 1747 reflections
*a* = 10.6611 (19) Å	θ = 2.2–23.5º
*b* = 10.3876 (18) Å	µ = 0.07 mm^−^^1^
*c* = 19.541 (3) Å	*T* = 295 (2) K
*V* = 2164.0 (6) Å^3^	Needle, colorless
*Z* = 4	0.34 × 0.24 × 0.18 mm

### Data collection

**Table:**

Bruker SMART APEX area-detector diffractometer	2138 independent reflections
Radiation source: fine-focus sealed tube	1733 reflections with *I* > 2σ(*I*)
Monochromator: graphite	*R*_int_ = 0.031
*T* = 295(2) K	θ_max_ = 26.0º
φ and ω scans	θ_min_ = 2.1º
Absorption correction: multi-scan(SADABS; Sheldrick, 1996)	*h* = −13→7
*T*_min_ = 0.977, *T*_max_ = 0.991	*k* = −12→12
8705 measured reflections	*l* = −22→23

### Refinement

**Table:**

Refinement on *F*^2^	Secondary atom site location: difference Fourier map
Least-squares matrix: full	Hydrogen site location: inferred from neighbouring sites
*R*[*F*^2^ > 2σ(*F*^2^)] = 0.057	H-atom parameters constrained
*wR*(*F*^2^) = 0.135	*w* = 1/[σ^2^(*F*_o_^2^) + (0.0666*P*)^2^ + 0.0883*P*] where *P* = (*F*_o_^2^ + 2*F*_c_^2^)/3
*S* = 1.06	(Δ/σ)_max_ < 0.001
2138 reflections	Δρ_max_ = 0.14 e Å^−^^3^
256 parameters	Δρ_min_ = −0.12 e Å^−^^3^
1 restraint	Extinction correction: none
Primary atom site location: structure-invariant direct methods	

### Special details

**Table:**

Geometry. All e.s.d.'s (except the e.s.d. in the dihedral angle between two l.s. planes) are estimated using the full covariance matrix. The cell e.s.d.'s are taken into account individually in the estimation of e.s.d.'s in distances, angles and torsion angles; correlations between e.s.d.'s in cell parameters are only used when they are defined by crystal symmetry. An approximate (isotropic) treatment of cell e.s.d.'s is used for estimating e.s.d.'s involving l.s. planes.
Refinement. Refinement of *F*^2^ against ALL reflections. The weighted *R*-factor *wR* and goodness of fit *S* are based on *F*^2^, conventional *R*-factors *R* are based on *F*, with *F* set to zero for negative *F*^2^. The threshold expression of *F*^2^ > σ(*F*^2^) is used only for calculating *R*-factors(gt) *etc*. and is not relevant to the choice of reflections for refinement. *R*-factors based on *F*^2^ are statistically about twice as large as those based on *F*, and *R*- factors based on ALL data will be even larger.

### Fractional atomic coordinates and isotropic or equivalent isotropic displacement parameters (Å^2^)

**Table:**

	*x*	*y*	*z*	*U*_iso_*/*U*_eq_	
O1	0.1548 (3)	0.9299 (3)	0.61714 (16)	0.0824 (9)	
O2	−0.0475 (3)	0.8225 (3)	0.80106 (17)	0.0857 (10)	
C1	0.1191 (4)	0.7133 (3)	0.70389 (19)	0.0548 (9)	
H1	0.0388	0.7453	0.6864	0.066*	
C2	0.0994 (4)	0.5757 (3)	0.72761 (18)	0.0529 (9)	
C3	−0.0047 (4)	0.5062 (4)	0.7085 (3)	0.0791 (12)	
H3	−0.0676	0.5465	0.6835	0.095*	
C4	−0.0181 (5)	0.3786 (4)	0.7256 (3)	0.0899 (15)	
H4	−0.0904	0.3352	0.7122	0.108*	
C5	0.0718 (5)	0.3135 (4)	0.7616 (3)	0.0782 (11)	
C6	0.1716 (4)	0.3836 (4)	0.7829 (3)	0.0769 (12)	
H6	0.2322	0.3439	0.8099	0.092*	
C7	0.1870 (4)	0.5121 (4)	0.7660 (2)	0.0706 (11)	
H7	0.2580	0.5558	0.7809	0.085*	
C8	0.0598 (6)	0.1716 (4)	0.7788 (3)	0.1081 (17)	
H8A	−0.0175	0.1569	0.8026	0.162*	
H8B	0.1289	0.1459	0.8072	0.162*	
H8C	0.0607	0.1221	0.7373	0.162*	
C9	0.2132 (4)	0.7156 (4)	0.64500 (19)	0.0607 (10)	
H9A	0.2951	0.6936	0.6632	0.073*	
H9B	0.1901	0.6487	0.6127	0.073*	
C10	0.2252 (4)	0.8403 (4)	0.6063 (2)	0.0606 (10)	
C11	0.3257 (4)	0.8509 (3)	0.55339 (19)	0.0609 (10)	
C12	0.3500 (5)	0.9686 (4)	0.5225 (3)	0.0853 (14)	
H12	0.3015	1.0398	0.5339	0.102*	
C13	0.4443 (5)	0.9817 (5)	0.4753 (3)	0.0933 (16)	
H13	0.4578	1.0616	0.4550	0.112*	
C14	0.5194 (5)	0.8798 (5)	0.4572 (2)	0.0795 (13)	
C15	0.4960 (5)	0.7631 (4)	0.4875 (2)	0.0800 (13)	
H15	0.5449	0.6923	0.4757	0.096*	
C16	0.4018 (5)	0.7487 (4)	0.5350 (2)	0.0738 (12)	
H16	0.3890	0.6686	0.5551	0.089*	
C17	0.6266 (6)	0.8948 (6)	0.4070 (3)	0.1075 (17)	
H17A	0.6484	0.8121	0.3886	0.161*	
H17B	0.6979	0.9308	0.4302	0.161*	
H17C	0.6015	0.9510	0.3705	0.161*	
C18	0.1605 (4)	0.8028 (3)	0.76181 (19)	0.0553 (9)	
H18A	0.2365	0.7687	0.7822	0.066*	
H18B	0.1803	0.8866	0.7428	0.066*	
C19	0.0632 (4)	0.8189 (3)	0.8167 (2)	0.0559 (9)	
C20	0.0996 (4)	0.8358 (3)	0.8894 (2)	0.0549 (9)	
C21	0.2152 (4)	0.7957 (4)	0.9149 (2)	0.0653 (10)	
H21	0.2747	0.7611	0.8854	0.078*	
C22	0.2420 (5)	0.8068 (4)	0.9838 (2)	0.0801 (13)	
H22	0.3194	0.7788	0.9999	0.096*	
C23	0.1575 (6)	0.8582 (4)	1.0291 (2)	0.0835 (12)	
C24	0.0430 (6)	0.9012 (4)	1.0035 (3)	0.0887 (14)	
H24	−0.0153	0.9381	1.0329	0.106*	
C25	0.0156 (4)	0.8894 (4)	0.9351 (2)	0.0752 (13)	
H25	−0.0617	0.9182	0.9192	0.090*	
C26	0.1894 (7)	0.8665 (6)	1.1037 (3)	0.1172 (19)	
H26A	0.1194	0.9016	1.1283	0.176*	
H26B	0.2611	0.9211	1.1097	0.176*	
H26C	0.2080	0.7820	1.1208	0.176*	

### Atomic displacement parameters (Å^2^)

**Table:**

	*U*^11^	*U*^22^	*U*^33^	*U*^12^	*U*^13^	*U*^23^
O1	0.094 (2)	0.0654 (17)	0.0881 (19)	0.0209 (16)	−0.0006 (18)	0.0156 (16)
O2	0.0433 (17)	0.115 (2)	0.099 (2)	0.0087 (15)	−0.0059 (16)	−0.0162 (19)
C1	0.048 (2)	0.0560 (19)	0.060 (2)	0.0067 (16)	−0.0107 (18)	0.0025 (16)
C2	0.050 (2)	0.0518 (19)	0.057 (2)	0.0026 (16)	−0.0078 (17)	−0.0037 (15)
C3	0.075 (3)	0.072 (2)	0.091 (3)	−0.006 (2)	−0.032 (2)	0.004 (2)
C4	0.088 (3)	0.072 (3)	0.110 (4)	−0.023 (2)	−0.024 (3)	−0.002 (3)
C5	0.089 (3)	0.0534 (18)	0.092 (3)	−0.005 (2)	0.003 (2)	0.005 (2)
C6	0.068 (3)	0.065 (2)	0.098 (3)	0.001 (2)	−0.014 (2)	0.021 (2)
C7	0.055 (2)	0.064 (2)	0.093 (3)	−0.0060 (18)	−0.019 (2)	0.009 (2)
C8	0.125 (4)	0.061 (2)	0.139 (4)	−0.010 (2)	0.002 (3)	0.010 (3)
C9	0.069 (3)	0.0511 (19)	0.061 (2)	0.0057 (17)	−0.0043 (19)	0.0020 (16)
C10	0.070 (3)	0.054 (2)	0.058 (2)	0.0094 (19)	−0.015 (2)	0.0019 (17)
C11	0.081 (3)	0.053 (2)	0.049 (2)	−0.0012 (19)	−0.0134 (19)	0.0049 (15)
C12	0.107 (4)	0.061 (2)	0.088 (3)	0.007 (2)	0.000 (3)	0.013 (2)
C13	0.115 (4)	0.068 (3)	0.097 (4)	−0.014 (3)	0.008 (3)	0.024 (3)
C14	0.099 (4)	0.081 (3)	0.059 (2)	−0.019 (3)	−0.003 (2)	0.000 (2)
C15	0.097 (4)	0.068 (2)	0.075 (3)	−0.002 (2)	0.011 (3)	−0.003 (2)
C16	0.100 (3)	0.053 (2)	0.068 (2)	−0.001 (2)	0.006 (3)	0.0071 (18)
C17	0.127 (4)	0.107 (4)	0.089 (3)	−0.028 (3)	0.017 (3)	−0.002 (3)
C18	0.047 (2)	0.0551 (19)	0.064 (2)	−0.0002 (16)	−0.0026 (18)	0.0019 (17)
C19	0.041 (2)	0.0504 (19)	0.076 (2)	0.0015 (16)	0.0002 (19)	−0.0025 (17)
C20	0.052 (2)	0.0413 (17)	0.072 (2)	−0.0100 (16)	0.0101 (19)	−0.0044 (16)
C21	0.066 (3)	0.066 (2)	0.064 (3)	0.003 (2)	0.002 (2)	−0.0019 (19)
C22	0.095 (4)	0.075 (3)	0.070 (3)	0.004 (3)	−0.002 (3)	0.008 (2)
C23	0.124 (3)	0.055 (2)	0.071 (3)	−0.027 (2)	0.016 (3)	−0.005 (2)
C24	0.113 (3)	0.067 (2)	0.086 (3)	−0.018 (2)	0.032 (3)	−0.025 (2)
C25	0.066 (3)	0.061 (2)	0.098 (4)	−0.007 (2)	0.013 (2)	−0.021 (2)
C26	0.176 (6)	0.103 (4)	0.072 (3)	−0.035 (4)	0.008 (4)	−0.010 (3)

### Geometric parameters (Å, °)

**Table:**

O1—C10	1.214 (4)	C13—C14	1.374 (7)
O2—C19	1.219 (5)	C13—H13	0.9300
C1—C2	1.517 (5)	C14—C15	1.371 (7)
C1—C9	1.527 (6)	C14—C17	1.514 (8)
C1—C18	1.530 (5)	C15—C16	1.375 (7)
C1—H1	0.9800	C15—H15	0.9300
C2—C7	1.368 (5)	C16—H16	0.9300
C2—C3	1.375 (6)	C17—H17A	0.9600
C3—C4	1.373 (6)	C17—H17B	0.9600
C3—H3	0.9300	C17—H17C	0.9600
C4—C5	1.369 (7)	C18—C19	1.501 (6)
C4—H4	0.9300	C18—H18A	0.9700
C5—C6	1.355 (7)	C18—H18B	0.9700
C5—C8	1.517 (6)	C19—C20	1.483 (6)
C6—C7	1.385 (6)	C20—C25	1.383 (6)
C6—H6	0.9300	C20—C21	1.393 (6)
C7—H7	0.9300	C21—C22	1.380 (6)
C8—H8A	0.9600	C21—H21	0.9300
C8—H8B	0.9600	C22—C23	1.371 (7)
C8—H8C	0.9600	C22—H22	0.9300
C9—C10	1.506 (5)	C23—C24	1.392 (8)
C9—H9A	0.9700	C23—C26	1.499 (8)
C9—H9B	0.9700	C24—C25	1.372 (8)
C10—C11	1.493 (6)	C24—H24	0.9300
C11—C16	1.384 (6)	C25—H25	0.9300
C11—C12	1.389 (6)	C26—H26A	0.9600
C12—C13	1.371 (7)	C26—H26B	0.9600
C12—H12	0.9300	C26—H26C	0.9600
			
C2—C1—C9	109.6 (3)	C15—C14—C13	117.7 (5)
C2—C1—C18	112.7 (3)	C15—C14—C17	120.5 (5)
C9—C1—C18	111.0 (3)	C13—C14—C17	121.8 (5)
C2—C1—H1	107.8	C14—C15—C16	121.3 (5)
C9—C1—H1	107.8	C14—C15—H15	119.3
C18—C1—H1	107.8	C16—C15—H15	119.3
C7—C2—C3	116.5 (4)	C15—C16—C11	121.3 (4)
C7—C2—C1	121.9 (3)	C15—C16—H16	119.3
C3—C2—C1	121.6 (3)	C11—C16—H16	119.3
C4—C3—C2	121.7 (4)	C14—C17—H17A	109.5
C4—C3—H3	119.2	C14—C17—H17B	109.5
C2—C3—H3	119.2	H17A—C17—H17B	109.5
C5—C4—C3	121.9 (4)	C14—C17—H17C	109.5
C5—C4—H4	119.0	H17A—C17—H17C	109.5
C3—C4—H4	119.0	H17B—C17—H17C	109.5
C6—C5—C4	116.2 (4)	C19—C18—C1	113.3 (3)
C6—C5—C8	121.4 (5)	C19—C18—H18A	108.9
C4—C5—C8	122.3 (5)	C1—C18—H18A	108.9
C5—C6—C7	122.5 (4)	C19—C18—H18B	108.9
C5—C6—H6	118.7	C1—C18—H18B	108.9
C7—C6—H6	118.7	H18A—C18—H18B	107.7
C2—C7—C6	121.1 (4)	O2—C19—C20	119.3 (4)
C2—C7—H7	119.5	O2—C19—C18	119.6 (4)
C6—C7—H7	119.5	C20—C19—C18	121.1 (3)
C5—C8—H8A	109.5	C25—C20—C21	117.5 (4)
C5—C8—H8B	109.5	C25—C20—C19	119.8 (4)
H8A—C8—H8B	109.5	C21—C20—C19	122.7 (3)
C5—C8—H8C	109.5	C22—C21—C20	120.5 (4)
H8A—C8—H8C	109.5	C22—C21—H21	119.7
H8B—C8—H8C	109.5	C20—C21—H21	119.7
C10—C9—C1	116.6 (3)	C23—C22—C21	121.7 (5)
C10—C9—H9A	108.1	C23—C22—H22	119.2
C1—C9—H9A	108.1	C21—C22—H22	119.1
C10—C9—H9B	108.1	C22—C23—C24	118.0 (5)
C1—C9—H9B	108.1	C22—C23—C26	120.0 (6)
H9A—C9—H9B	107.3	C24—C23—C26	122.0 (5)
O1—C10—C11	120.6 (3)	C25—C24—C23	120.5 (5)
O1—C10—C9	121.3 (4)	C25—C24—H24	119.7
C11—C10—C9	118.1 (3)	C23—C24—H24	119.7
C16—C11—C12	116.9 (4)	C24—C25—C20	121.8 (5)
C16—C11—C10	123.0 (3)	C24—C25—H25	119.1
C12—C11—C10	120.0 (4)	C20—C25—H25	119.1
C13—C12—C11	121.1 (5)	C23—C26—H26A	109.5
C13—C12—H12	119.4	C23—C26—H26B	109.5
C11—C12—H12	119.4	H26A—C26—H26B	109.5
C12—C13—C14	121.6 (4)	C23—C26—H26C	109.5
C12—C13—H13	119.2	H26A—C26—H26C	109.5
C14—C13—H13	119.2	H26B—C26—H26C	109.5
			
C9—C1—C2—C7	75.1 (5)	C12—C13—C14—C15	0.7 (8)
C18—C1—C2—C7	−49.1 (5)	C12—C13—C14—C17	−177.8 (5)
C9—C1—C2—C3	−101.4 (4)	C13—C14—C15—C16	−0.8 (7)
C18—C1—C2—C3	134.5 (4)	C17—C14—C15—C16	177.7 (5)
C7—C2—C3—C4	−1.7 (7)	C14—C15—C16—C11	0.9 (7)
C1—C2—C3—C4	174.9 (4)	C12—C11—C16—C15	−0.8 (6)
C2—C3—C4—C5	−0.8 (8)	C10—C11—C16—C15	−178.0 (4)
C3—C4—C5—C6	3.5 (8)	C2—C1—C18—C19	−64.7 (4)
C3—C4—C5—C8	−178.0 (5)	C9—C1—C18—C19	171.9 (3)
C4—C5—C6—C7	−3.8 (8)	C1—C18—C19—O2	−36.5 (5)
C8—C5—C6—C7	177.7 (5)	C1—C18—C19—C20	145.9 (3)
C3—C2—C7—C6	1.5 (7)	O2—C19—C20—C25	−17.7 (5)
C1—C2—C7—C6	−175.2 (4)	C18—C19—C20—C25	159.9 (3)
C5—C6—C7—C2	1.4 (7)	O2—C19—C20—C21	160.1 (4)
C2—C1—C9—C10	168.5 (3)	C18—C19—C20—C21	−22.3 (5)
C18—C1—C9—C10	−66.3 (4)	C25—C20—C21—C22	1.5 (6)
C1—C9—C10—O1	−6.9 (6)	C19—C20—C21—C22	−176.3 (4)
C1—C9—C10—C11	173.5 (3)	C20—C21—C22—C23	−0.5 (7)
O1—C10—C11—C16	−174.6 (4)	C21—C22—C23—C24	−0.9 (7)
C9—C10—C11—C16	5.0 (6)	C21—C22—C23—C26	178.9 (5)
O1—C10—C11—C12	8.3 (6)	C22—C23—C24—C25	1.4 (7)
C9—C10—C11—C12	−172.1 (4)	C26—C23—C24—C25	−178.4 (4)
C16—C11—C12—C13	0.8 (7)	C23—C24—C25—C20	−0.4 (7)
C10—C11—C12—C13	178.0 (4)	C21—C20—C25—C24	−1.0 (6)
C11—C12—C13—C14	−0.7 (8)	C19—C20—C25—C24	176.9 (4)

### Hydrogen-bond geometry (Å, °)

**Table:**

*D*—H···*A*	*D*—H	H···*A*	*D*···*A*	*D*—H···*A*
C7—H7···O2^i^	0.93	2.46	3.381 (5)	171 (1)
C18—H18A···O2^i^	0.97	2.52	3.460 (5)	164 (1)

Symmetry codes: (i) *x*+1/2, −*y*+3/2, *z*.
